# Case report: Fluoroscopic-assisted closed reduction and minimally invasive femoral capital physeal fracture repair in four calves

**DOI:** 10.3389/fvets.2022.970220

**Published:** 2022-09-26

**Authors:** Avery F. Loyd, Dane M. Tatarniuk, Jaron H. Naiman, Paul T. Merkatoris, Jarrod R. Troy

**Affiliations:** ^1^Department of Veterinary Clinical Sciences, Iowa State University, Ames, IA, United States; ^2^Highline Veterinary Orthopedics, Lakeville, MN, United States; ^3^VCA Animal Specialty and Emergency Center, Los Angeles, CA, United States; ^4^Department of Surgical Sciences, University of Wisconsin-Madison, Madison, WI, United States

**Keywords:** calves, femur, capital, physeal, fracture, fluoroscopy

## Abstract

**Objective:**

To describe a minimally invasive osteosynthesis (MIO) femoral capital physeal fracture (FCPF) repair technique using multiple smooth Steinmann pins in four calves.

**Study design:**

Case series.

**Animal:**

Four calves (< 60 days of age).

**Methods:**

Medical records at a single referral hospital were searched for calves that had minimally invasive osteosynthesis (MIO) femoral capital physeal fracture (FCPF) repair performed using multiple Steinmann pins between 2020 and 2021. Calves receiving alternative repair, euthanasia without repair, or > 60 days of age were excluded. Medical records were reviewed together the following information: inciting FCPF cause, patient signalment, clinical sign duration pre-admission, history of dystocia, and any pre-admission treatment. Preoperative parameters collected included packed cell volume (PCV), serum total solids (TS), additional bloodwork when available, peripheral blood glucose, antimicrobial therapy, and analgesic medications. Preoperative coxofemoral radiographic images of all calves were obtained.

**Results/outcome:**

Four calves were presented with severe hind limb lameness from varying etiologies. FCPF was diagnosed in all calves *via* radiograph. All FCPFs were repaired with an MIO repair technique using multiple Steinmann pins. Intraoperative fracture reduction and fixation were deemed appropriate by the attending surgeon with the use of fluoroscopy. Postoperatively, all calves retained normal weight bearing and were ambulating. One calf died postoperatively due to an unrelated comorbidity (severe bronchopneumonia and hyperkalemia). The three remaining calves survived to hospital discharge and were ambulating normally with an adequate range of motion at the time of discharge. Long-term follow-up reports were available for two cases, which revealed long-term survival at 210- and 146-days. Owners reported good ambulation, and one of the calves was placed in the show ring and was performing. However, one calf was lost to long-term follow-up.

**Conclusion:**

MIO FCPF repair with multiple Steinmann pins, previously described in small animal species, can be implemented for FCPF repair in young calves.

**Clinical impact:**

This case series provides a foundation for minimally invasive osteosynthesis technique translation to large animal juveniles and reports an alternative MIO technique for capital physeal closed fracture repair in calves.

## Introduction

Femoral capital physeal fracture (FCPF) configurations are common in calves and foals secondary to dystocia or external injury (1–5), Traditionally, large animal FCPF repair involves an open approach over the femoral neck and the coxofemoral joint followed by internal fixation with smooth Steinmann pins, cannulated screws, modified dynamic hip-screw systems, or cortical lag screws (1–6). Prognosis followed by repair varies by age and bodyweight increase with a good prognosis in calves aged < 12 months but fair prognosis in calves aged > 12 months (3–5). Minimally invasive osteosynthesis (MIO) techniques for FCPF repair in human, feline, and canine patients have been reported with reduced morbidity and postoperative complications compared to traditional open approaches (7–9). While MIO principles are limited in adults of large animal species due to size, neonatal application may be viable, leading to reduced morbidity and postoperative complications in FCPF cases. This case series describes unilateral FCPF MIO repair with multiple, transphyseal, smooth Steinmann pins in four calves.

## Materials and methods

The Iowa State University Lloyd Veterinary Medical Center (ISU LVMC) database was screened for calves with unilateral FCPF repair using an MIO technique with smooth Steinman pin internal fixation between 1 January 2020 and 1 July 2021. Calves receiving alternative repair, euthanasia without repair, or > 60 days of age were excluded. Medical records were reviewed to obtain the following information: inciting FCPF cause, patient signalment, clinical sign duration pre-admission, history of dystocia, and any pre-admission treatment. Preoperative parameters collected included packed cell volume (PCV), serum total solids (TS), peripheral blood glucose, additional bloodwork when available, antimicrobial therapy, and analgesic medications. Preoperative coxofemoral radiograph images were obtained in all calves, including flexed limb ventral-dorsal, extended limb ventral-dorsal, and lateral-medial views. All radiographs were evaluated by a board-certified radiologist *via* a teleradiology consulting service (Vet-Ct©, Florida, USA).

Intraoperative and postoperative parameters collected as available included antimicrobial therapy, analgesic administration, anesthetic duration, surgical duration, and hospitalization duration. Other data collected as available included serum electrolytes, peripheral total white blood cell count (t-WBC), plasma fibrinogen, blood urea nitrogen (BUN), serum creatinine, aspartate aminotransferase (AST), and gamma-glutamyl transferase (GGT). Patient comorbidity data included corresponding comorbidity diagnosis and treatment. Short-term and long-term outcome information was collected for all calves. Short-term follow-up was defined as survival to hospital discharge and long-term follow-up >4 months postoperatively. Owners were contacted by phone call to assess long-term survival, owner perception of limb function, and performance with the intended use.

### Surgical description

**Figure 1:**
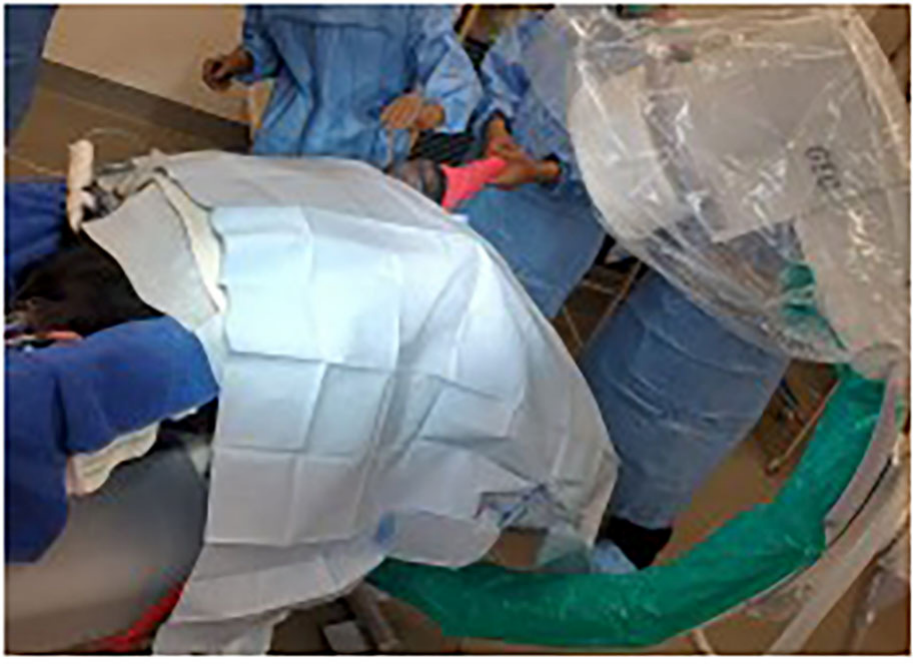
Visualization of patient position. Dorsal recumbency at the level of the withers with pelvis tilted toward a right lateral recumbency. The back half of the calf is on a radiolucent plexiglass table extension that allows intraoperative fluoroscopic imaging.

**Figure 2:**
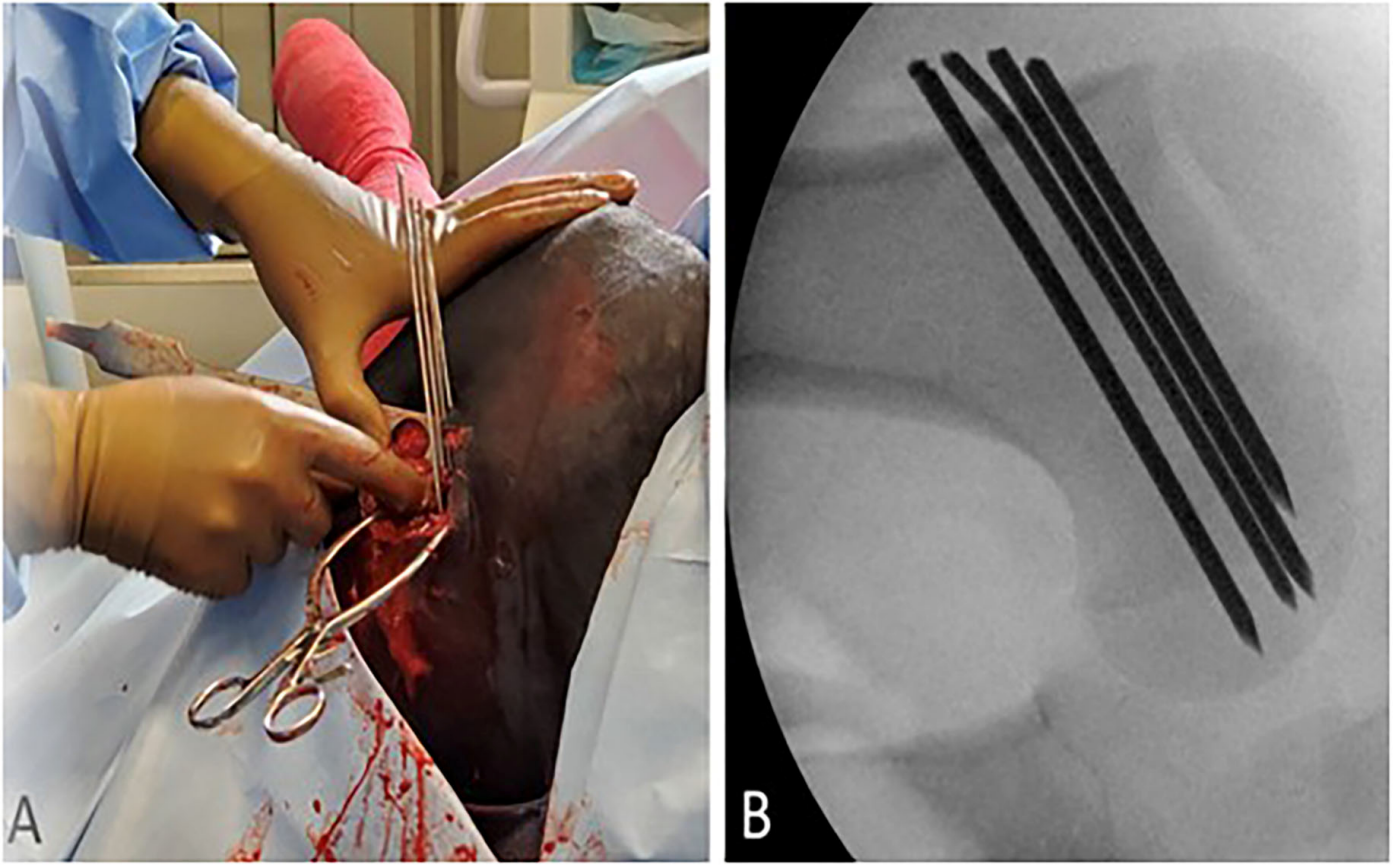
**(A)** External visualization of four smooth Steinmann pins placed in a parallel orientation through a skin incision on the lateral proximal femur. **(B)** Intraoperative fluoroscopic view of final Steinmann pin fixation through the left proximal femoral physis.

Preoperative medications included 1 mg/kg meloxicam (Meloxicam tablets, Zydus Pharmaceuticals, Pennington, NJ) orally and 40 mg/kg subcutaneous florfenicol (Nuflor, Florfenicol Injectable Solution, Merck & Co., Inc., Kenilworth, NJ). Anesthetic premedication was 0.05 mg/kg intravenous hydromorphone (Hydromorhpone HCl injection, West-Ward, Eatontown, NJ) and 0.2 mg/kg intravenous midazolam (Midazolam HCl injection, Athenex Pharmaceutical Division, LLC, Buffalo, NY) followed by 2.5 mg/kg intravenous propofol (Propofol Injectable Emulsion, Pfizer Inc., Ney York, NY). Following induction, a 0.1 mg/kg morphine (Duramorph, Preservative-Free Morphine Sulfate Injection, West-Ward, Eatontown, NJ) lumbosacral epidural was performed with inhalant isoflurane for anesthetic maintenance (Fluriso, Isoflurane, VetOne, Boise, ID). Calves were placed in dorsal recumbency only or in dorsal recumbency with the caudal 1/3 of the body angled laterally with the fractured limb uppermost. The lumbar-pelvic region was placed on a radiolucent plexiglass fluoroscopy table. The limb was clipped and aseptically prepared from the pelvis down to the digits. The fluoroscopy C-arm was placed so that the affected proximal femoral capitus, the neck, and the coxofemoral joint were visible fluoroscopically on ventral-dorsal and lateral-medial images. Images were acquired by repositioning the lumbar-pelvic region between dorsal or lateral recumbency as needed with the generator that is dorsal or lateral to the patient (Figure 1). Fracture reduction was achieved by distal limb caudal extension with lateral to medial manual force applied to the greater trochanter. Limb adduction or internal rotation was aided in the reduction for some cases. Static and continuous fluoroscopy was utilized to assess fracture reduction and implant placement. A smooth Steinman pin insertion site was determined by placing the pin 2–4 cm distal to the palpable aspect of the greater trochanter such that Steinman pin placement would engage the femoral capitus and the neck (Figure 2). At this site, a 6-cm incision was created to the lateral femoral cortex through the skin, the subcutaneous tissues, and the biceps femoris muscle. Two 1.2 mm Kirschner (K-) wires were placed adjacent to the cranial and caudal limbs for Steinmann pin insertion guidance. Steinman pin size (range, 2.3–4.0 mm) was determined by the attending surgeon. The Steinman pin was then placed at the proposed insertion point between the 2 K-wires in an orientation that would engage both the femoral capitus and the neck during anatomic reduction. All Steinman pins were placed with a power driver using Jacob's chuck attachment. The initial Steinman pin was seated into the femoral neck without crossing the physis and any additional reduction was performed. The Steinman pin was then advanced across the physis engaging the femoral capitus until the pin was secure but did not appear to penetrate the articular margin fluoroscopically. Subsequent Steinman pins were inserted parallel or slightly divergent to the initial Steinman pin for additional stability (Figure 2B). The distal pin ends were transected using pin-cutters or bolt cutters (H. K. Porter Center Cut Bolt Cutters, Apex Tool Group, LLC, Spark, MD) so that the distal extent was contiguous with the biceps femoris m. epimysium. Reduction and implant security were evaluated with fluoroscopy prior to incision closure. The epimysium and subcutaneous tissues were closed in a simple continuous pattern with 2-0 poliglecaprone 25. The skin was closed either with a simple continuous pattern using 0 poliglecaprone 25 or skin staples. All incisions were coated with an aluminum-based wound barrier product (AluSpray, Neogen Vet, Lexington, KY), and hind limb hobbles were applied above the fetlocks to reduce “splay-leg” risk or slipping postoperatively (10). Instructions included hobble maintenance for 10–14 days postoperatively with 30-day small pen confinement.

## Results

**Figure 3:**
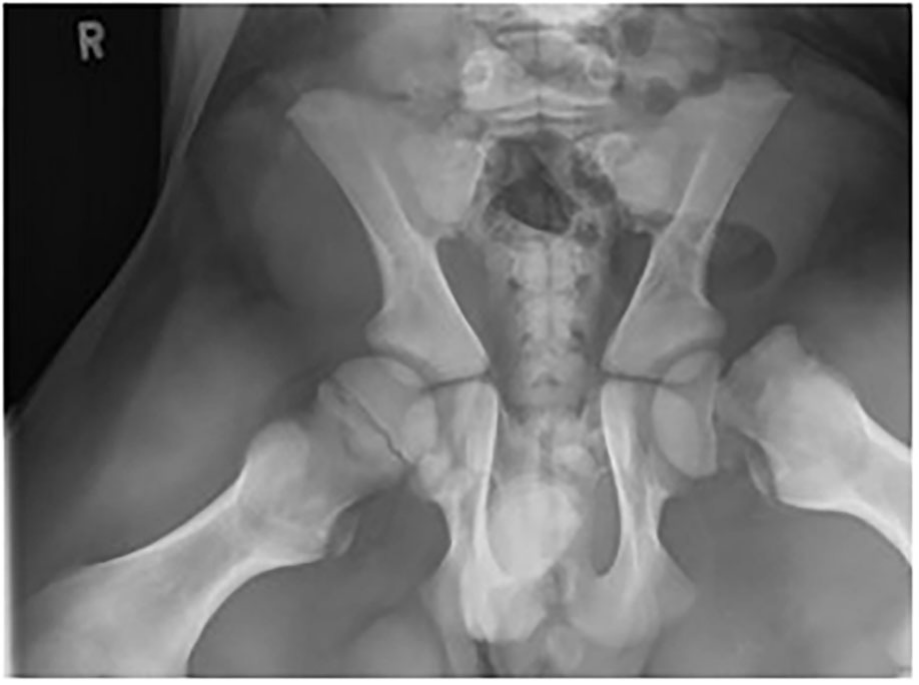
Ventral-dorsal projection of the pelvis demonstrating a type 1 Salter-Harris (capital physeal) fracture in the left proximal femoral physis (case 1).

Four calves were presented to the ISU LVMC for FCPF that received MIO Steinman pin internal fixation repair between 2020 and 2021 with individual case information and outcomes recorded below. Radiographically FCPF was diagnosed in the lame limb with no evidence of acetabular or femoral diaphyseal fracture (Figure 3) in all cases. All calves received the same preoperative medications, general anesthesia, and surgical procedure as described above. Steinmann pin sizes were unique to each calf and were recorded below.

### Case #1

**Figure 4:**
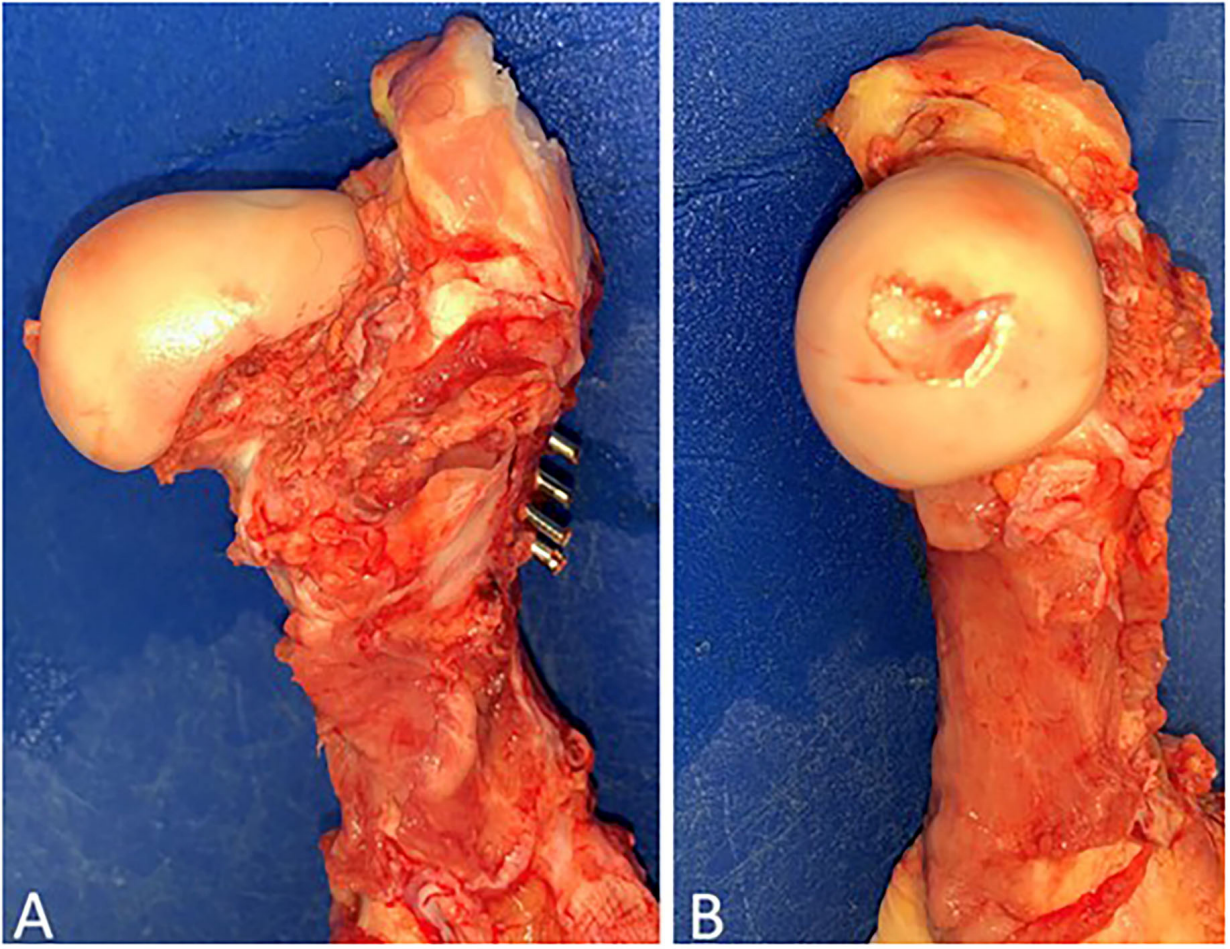
Gross post-mortem exam of the left hind proximal femur of case 1. Note adequate reduction and fixation without the presence of articular compromise. **(A)** Cranial-caudal and **(B)** Medial-lateral.

A 1-day-old mixed breed beef bull (BWT = 45 kg) was presented for left hind limb lameness lasting for 18 h after parturition. There was a history of dystocia with the owner delivering the calf through a ratchet-style calf puller. The owner reported the calf to be standing and nursing normally but to have toe-touching lameness on the left hind limb. On admission, partial weight-bearing lameness on the left hind limb was observed with palpable medial patellar luxation. The remaining physical exam findings were unremarkable. Serum total protein was 7.1 g/dL, indicating adequate colostrum absorption (11). No other preoperative bloodwork was recorded. FCPF MIO Steinmann pin repair, described above, was performed using four Steinman pins as follows, one 3/32” (2.3 mm) Steinman pin with three subsequent 7/64” (2.7 mm) Steinman pins. The surgical time was 101 min. Subjectively, anesthetic recovery was prolonged, but the recovery time was not recorded. Postoperatively, the left hind limb of the calf was weight bearing, and the calf was ambulating around the stall but appeared dull. Postoperative bloodwork revealed hyperglycemia 600 mg/dL (range: 40–100) and hyperkalemia 7.0 mEq/L (range: 3.7–5.3). The calf was treated with 0.2 U/kg IV regular insulin (Humulin R, Regular Insulin Human Injection, Lilly USA, LLC, Indianapolis, IN) and 0.5 ml/kg IV 50% dextrose solution bolus (Dextrose 50% injection, VetOne, Boise, ID). Thoracoabdominal ultrasonography revealed bilateral B-lines, no free peritoneal fluid, and an intact urinary bladder. The calf was hospitalized and maintained on intravenous fluid therapy at 60 ml/kg/day using 0.9% NaCl (0.9% Sodium Chloride Solution Injection, B. Braun, Medical Inc., Bethlehem, PA). Glucose and potassium were monitored every 2 h, with repeat insulin administration as required. Seven hours postoperatively, potassium was 4.8 mmol/L and glucose was 300 mg/dL. Eight hours postoperatively, the calf underwent cardiopulmonary arrest with unsuccessful cardiopulmonary resuscitation. Postmortem evaluation diagnosed severe, acute, diffuse, and fibrinopurulent bronchopneumonia. During the postmortem evaluation, the left femur and the coxofemoral joint were dissected from the cadaver. The left FCPF MIO repair with adequate anatomic reduction appeared to be appropriate on gross observation (Figure 4). All pins had satisfactory purchase within the femoral epiphysis and no articular penetration.

### Case #2

A 21-day-old mixed breed beef bull (BWT = 55 kg) was presented with a 20-day history of left hind limb lameness. History included dystocia resolved through cesarean (C-)section delivery, and left hind limb non-weight bearing lameness was observed the following day. The owner treated the calf with transdermal flunixin meglumine for 20 days before hospital admission. On admission, left hind limb non-bearing lameness, left gluteal muscle atrophy, and right tarsal varus angular deformity were observed. The remaining physical exam findings were unremarkable. FCPF MIO Steinmann pin repair was performed using three 1/8” (3.2 mm) Steinman pins with a surgical time of 82 min. Postoperatively, the calf was bearing weight on the left hind limb and ambulating with hobbles. Meloxicam (1 mg/kg PO q24h) was prescribed for 7 days postoperatively. The calf was discharged alive from the hospital 2 days postoperatively. Postoperative recheck and radiographs were recommended at 6 weeks. The owner did not return for recheck due to time constraints and did not report if the calf was doing well. At 210 days postoperatively, the owner was contacted through telephone for long-term follow-up. Reportedly, the calf was bearing weight on all limbs, displaying adequate body weight increase, and ambulating well but with a mild, mechanical-like gait according to the owner. The right tarsal varus remained but did not appear to be causing a problem. The owner was satisfied with the surgical repair but elected not to enter the calf in show competitions.

### Case #3

A 19-day-old Maine -Anjou heifer calf (BWT = 62.5 kg) was presented for chronic left hind limb lameness of indeterminant duration. On admission, the calf was non-weight bearing on the left hind limb with otherwise unremarkable physical exam findings. FCPF MIO Steinmann pin repair was performed using three 1/8” (3.2 mm) Steinman pins with a surgical time of 52 min. Postoperatively, the calf was bearing full weight and ambulating comfortably with hobbles. Meloxicam (1 mg/kg PO q24h) was prescribed for 7 days, and the calf was discharged alive from the hospital 5 days after surgery. Fourteen days after hospital discharge the owner reported incisional drainage but no lameness. It was recommended that skin sutures be removed and ceftiofur 6.6 mg/kg be administered subcutaneously (Excede, Ceftiofur Crystalline Free Acid sterile suspension, Zoetis Inc, Parsippany, NJ). Nineteen days postoperatively, telephonic follow-up with the owner revealed reduced incisional drainage. At that time, a 6-weeks recheck with radiographs was recommended but no appointment was scheduled. At 146 days postoperatively, the owner was contacted *via* telephone for long-term follow-up. The owner reported normal weight bearing with no appreciable lameness. The owner reported that the calf was ~100 lbs less in body weight compared to cohort calves but believed genetics to be the cause of the reduced weight. The owner was satisfied with the outcome of the FCPF repair. The calf was reportedly shown 8 times, had won 3 times, and had won the local county fair reserved champion feeder calf award. The date of the first show competition after hospital discharge was unknown by the owner.

### Case #4

A 60-day-old mixed breed beef bull (BWT = 145 kg) was presented with a 24-h history of acute, right hind limb lameness. No prior medications or therapy were performed. On admission, a partial weight-bearing right hind limb lameness was observed but the rest of the physical examination was unremarkable. FCPF MIO Steinmann pin repair was performed using four 5/32“ (4.0 mm) Steinman pins with a surgical time of 85 min. In this case, due to biceps femoris m. size and Steinman pin diameter, the distal extent of the Steinman pin could not be transected contiguous with the lateral surface of the biceps femoris muscle. The remaining distal pin length extended ~1–2 mm past biceps femoris m. epimysium but could be covered entirely with subcutaneous tissues. After skin closure, the pin ends were palpable but the skin did not appear tight or stretched from the pin ends. After anesthetic recovery, the calf was ambulating around the stall with hobbles and was fully bearing weight on the operated limb. Postoperative meloxicam (1 mg/kg body weight PO q 24 h) was prescribed for 7 days and the calf was discharged 2 days later. Fourteen days postoperatively, the calf developed acute lameness on the repaired limb. The owner reported that the calf would stand with the left hind limb fully extended while exhibiting non-weight bearing lameness on the right hind limb. Recommendations included meloxicam administration with re-evaluation at the owner's earliest convenience. The calf was readmitted 22-days later for evaluation. The calf was moderately right hind limb lameness but was bearing weight. The surgical site was dry and clean but raised about 1–2 cm. No incisional discharge was observed. Adequate and symmetrical muscling of the gluteal and quadriceps muscles were noted. Radiographs revealed minimal callus formation and no Steinman pin penetration into the coxofemoral articular margin. Surgical site ultrasonography showed no fluid accumulation or edema. Lameness was suspected to be secondary to distal Steinman pin irritation of the subcutaneous tissues. The owners were instructed that the calf to be confined to a small, dry pen for 2–4 more weeks with meloxicam (1 mg/kg PO q 24 h) prescribed as needed. A recheck appointment was recommended 6 weeks later with repeated radiography, but the owner along with the calf did not appear. Phone communication was attempted with the owner but case #4 was lost to follow-up.

## Discussion

This case series reported MIO FCPF repair in young calves using multiple Steinmann pins. FCPF repair in calves is internal fixation through an incision over the fracture site (1–6). Open approaches allowed fracture visualization for reduction and implant accessibility but required large incisions with associated tissue dissection (1–6). Comparatively, MIO FCPF repair can result in fracture reduction, appropriate stabilization, smaller incisions, fewer complications, and reduced morbidity (7–9). Despite these benefits, translating MIO FCPF repair to adult cattle is unattainable due to patient size and the strength of available implants (7–9). However, these benefits could be achieved in young calves due to their smaller size. In the cases reported here, FCPF internal fixation was achieved using multiple Steinmann pins as they reportedly preserve physeal integrity permitting continued longitudinal growth compared to other implants (1, 3, 5, 12). Although stabilization was achieved in these cases, comorbidities and patient size played a role in the overall outcome. Case #1 died subsequently due to hyperkalemia with pleuropneumonia diagnosed on necropsy. In this case, longer medical stabilization might have been helpful, but the calf appeared systemically stable with normal bronchovesicular sounds bilaterally. Additional preoperative bloodwork would have been valuable in this case but was frequently declined by the owners in these cases. In case #4, it was felt that the bodyweight impacted the overall outcome, as this calf was more than 80 kg heavier than the other calves. Larger Steinmann pins were used in this case due to a perceived risk of implant failure with smaller pin sizes. Subsequently, bolt cutters had to be used to transect the pins instead of the pin cutters used for cases 1–3. These bolt cutters could not fit within the incision to transect the distal pins contiguously with biceps femoris m. epimysium. Instead, 1–2 mm of Steinmann pin was left to remain within the subcutaneous tissue. This could have caused the postoperative lameness observed 22 days postoperatively. One possible solution was planning for a second surgery involving pin removal following callus formation. Another option would be pin transection with a sterile hacksaw. Future research directions could include determining patient size/bodyweight or implant size breakpoints that render large animal MIO FCPF repair impractical.

The MIO principles emphasized minimal soft tissue dissection and smaller incisions (7). Initially, percutaneous Steinman pin insertion was attempted in case #1; however, bovid skin thickness and muscle mass made this challenging for appropriately sized implants. A 6-cm skin incision was created instead of an individual Steinman pin stab incision in the following cases, which allowed close pin approximation. This could possibly reduce the benefits of MIO when compared to percutaneous insertion; however, the incision was smaller than reported craniolateral approaches (1–6). The FCPF itself was left undisturbed per MIO principles and reduced complications with an open fracture site. A challenge in these cases was predicting the number of pins and size before the start of the procedure. Further *in vitro* and clinical studies were indicated to assess the optimal size and the number of pins. Future mechanical studies could have evaluated the strength of multiple pins and size configurations compared to single or multiple lag screws.

This case series reports the use of MIO FCPF repair previously reported in small animal species for young bovids (7–9). The long-term outcome for return to intended use and performance as a show animal is possible. Prognosis remained uncertain due to a small case series population and it did appear that comorbidity and patient bodyweight play noteworthy roles in outcome following MIO FCPF. It would have been very helpful for postoperative radiographs and examination but could not be accomplished due to the animals not returning for re-evaluation. However, patient comorbidity and bodyweight could greatly impact patient outcomes following MIO FCPF repair with Steinmann pins.

## Data availability statement

The original contributions presented in the study are included in the article/supplementary material, further inquiries can be directed to the corresponding author/s.

## Author contributions

AL acquired the information from each individual case information, revised multiple drafts with conceptualization, and interpretation of all information. PM, DT, and JT performed the surgeries, acquired individual case information, and revised multiple drafts. JN performed the surgeries and aided in multiple draft revisions. All authors contributed to the article and approved the submitted version.

## Conflict of interest

The authors declare that the research was conducted in the absence of any commercial or financial relationships that could be construed as a potential conflict of interest.

## Publisher's note

All claims expressed in this article are solely those of the authors and do not necessarily represent those of their affiliated organizations, or those of the publisher, the editors and the reviewers. Any product that may be evaluated in this article, or claim that may be made by its manufacturer, is not guaranteed or endorsed by the publisher.
